# Oligogenic Effects of 16p11.2 Copy-Number Variation on Craniofacial Development

**DOI:** 10.1016/j.celrep.2019.08.071

**Published:** 2019-09-24

**Authors:** Yuqi Qiu, Thomas Arbogast, Sandra Martin Lorenzo, Hongying Li, Shih C. Tang, Ellen Richardson, Oanh Hong, Shawn Cho, Omar Shanta, Timothy Pang, Christina Corsello, Curtis K. Deutsch, Claire Chevalier, Erica E. Davis, Lilia M. lakoucheva, Yann Herault, Nicholas Katsanis, Karen Messer, Jonathan Sebat

**Affiliations:** 1 Division of Biostatistics and Bioinformatics, Department of Family Medicine and Public Health, University of California, San Diego, La Jolla, CA 92093, USA; 2 Center for Human Disease Modeling, Duke University, Durham, NC 27701, USA; 3 Institut de Génétique et de Biologie Moléculaire et Cellulaire, Université de Strasbourg, Illkirch, France; 4 Centre National de la Recherche Scientifique, UMR7104, Illkirch, France; 5 Institut National de la Santé et de la Recherche Médicale, U964, Illkirch, France; 6 Department of Psychiatry, University of California, San Diego, La Jolla, CA 92093, USA; 7 Department of Electrical Engineering, University of California, San Diego, La Jolla, CA 92093, USA; 8 Rady Children’s Hospital, San Diego, CA 92123, USA; 9 Eunice Kennedy Shriver Center UMMS, Charlestown and Worcester, MA, USA; 10 Beyster Center for Genomics of Psychiatric Diseases, University of California, San Diego, La Jolla, CA 92093, USA; 11 Institute for Genomic Medicine, University of California, San Diego, La Jolla, CA 92093, USA; 12 Department of Cellular and Molecular Medicine, University of California, San Diego, La Jolla, CA 92093, USA; 13 Department of Pediatrics, University of California, San Diego, La Jolla, CA 92093, USA; 14 These authors contributed equally; 15 Senior author; 16 Lead Contact

## Abstract

A copy-number variant (CNV) of 16p11.2 encompassing 30 genes is associated with developmental and psychiatric disorders, head size, and body mass. The genetic mechanisms that underlie these associations are not understood. To determine the influence of 16p11.2 genes on development, we investigated the effects of CNV on craniofacial structure in humans and model organisms. We show that deletion and duplication of 16p11.2 have “mirror” effects on specific craniofacial features that are conserved between human and rodent models of the CNV. By testing dosage effects of individual genes on the shape of the mandible in zebrafish, we identify seven genes with significant effects individually and find evidence for others when genes were tested in combination. The craniofacial phenotypes of 16p11.2 CNVs represent a model for studying the effects of genes on development, and our results suggest that the associated facial gestalts are attributable to the combined effects of multiple genes.

## In Brief

Using 3D morphometric imaging, Qiu et al. demonstrate that large copy-number variants (CNVs) of 16p11.2 have significant effects on craniofacial structure that are conserved in humans and model organisms, and they demonstrate that these craniofacial phenotypes are attributable to the dosage effects of multiple genes within the CNV region.

## Graphical Abstract

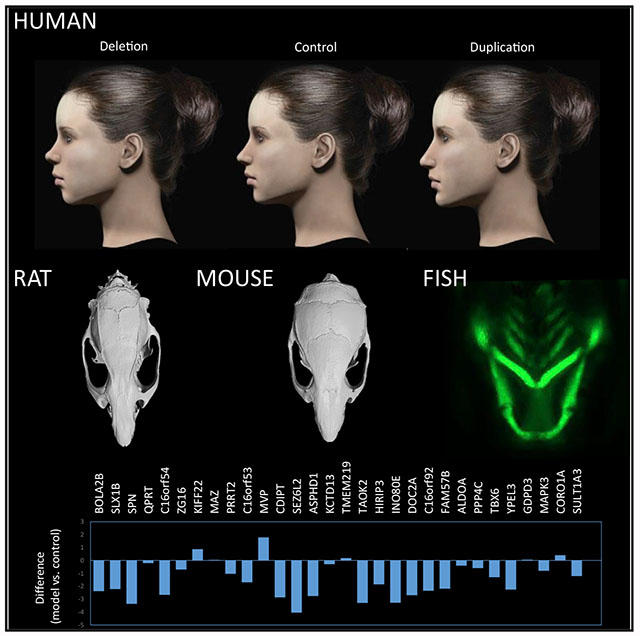

## INTRODUCTION

Recent technological advances in genomics have facilitated the discovery of scores of new genetic disorders that have a complex and variable clinical presentation (Malhotra and Sebat, 2012). Unlike Down syndrome (Roizen and Patterson, 2003) and Williams syndrome (Ewart et al., 1993), which have a distinguishable constellation of clinical features and facial gestalts, these new genetic disorders are notable for their lack of a clear pattern of congenital anomalies or dysmorphic features (Nevado et al., 2014). A major exemplar are the reciprocal copy-number variants (CNVs) of 16p11.2 (BP4-BP5, OMIM: 611913 and 614671). Deletion (Miller et al., 1993) and duplication (D’Angelo et al., 2016) of 30 genes are associated with variable degrees of cognitive impairment, epilepsy, and psychiatric disorders, including autism spectrum disorder and schizophrenia. We and others have shown that the dosage of 16p11.2 has quantitative effects on development, in particular morphometric traits, such as head circumference (McCarthy et al., 2009; Shinawi et al., 2010) and BMI (D’Angelo et al., 2016). Deletions are associated with greater head size and BMI, while duplications are associated with smaller head size and BMI. In addition, a variety of craniofacial anomalies have been reported in a subset of cases (Bijlsma et al., 2009; Rosenfeld et al., 2010; Shinawi et al., 2010), but a characteristic pattern of dysmorphic features has not been described. Thus, the influence of CNV on psychiatric and morphometric traits alike is complex, and the underlying genetic mechanisms are not understood.

Elucidating the genetic mechanisms through which CNVs influence development requires rigorous analysis of quantitative phenotype data in humans and the establishment of model systems in which the genetic mechanisms are conserved. Craniofacial development, in particular, is controlled by genetic mechanisms that are conserved across species (Schilling, 1997). The effect of genes on the human face is of interest, therefore, because craniofacial structure represents developmental phenotypes that are experimentally tractable in model organisms and that could provide insights into disease mechanisms. Effects of 16p11.2 CNV on development of the brain and head have been reported in both mouse (Arbogast et al., 2016; Horev et al., 2011) and zebrafish (Golzio et al., 2012), and multiple genes have been demonstrated to influence brain development, including KCTD13, MAPK3, and MVP (Arbogast et al., 2019; Escamilla et al., 2017; Golzio et al., 2012). We hypothesize that a precise morphometric characterization of patients could help to further illuminate how 16p11.2 genes influence embryonic development.

The application of 3D imaging provides detailed quantitative analysis of surface features, enabling more precise measurements of the shape of the head and face. Application of this approach has facilitated the finer characterization of genetic syndromes with characteristic craniofacial features (Hammond, 2007; Hammond et al., 2014). Application of this technology in non-syndromic and complex genetic disorders has the potential to elucidate the effect of genes on craniofacial development. By three-dimensional image analysis of surface features in human, rat, and mouse and the dissection of single gene effects in zebrafish, we show that the copy number of 16p11.2 has strong effects on craniofacial structure that are conserved across species, and the facial features associated with each disorder are attributable to the oligogenic effects of multiple genes.

## RESULTS

### Reciprocal Deletion and Duplication of 16p11.2 Have Mirror Effects on Craniofacial Structure

**Figure 1.**
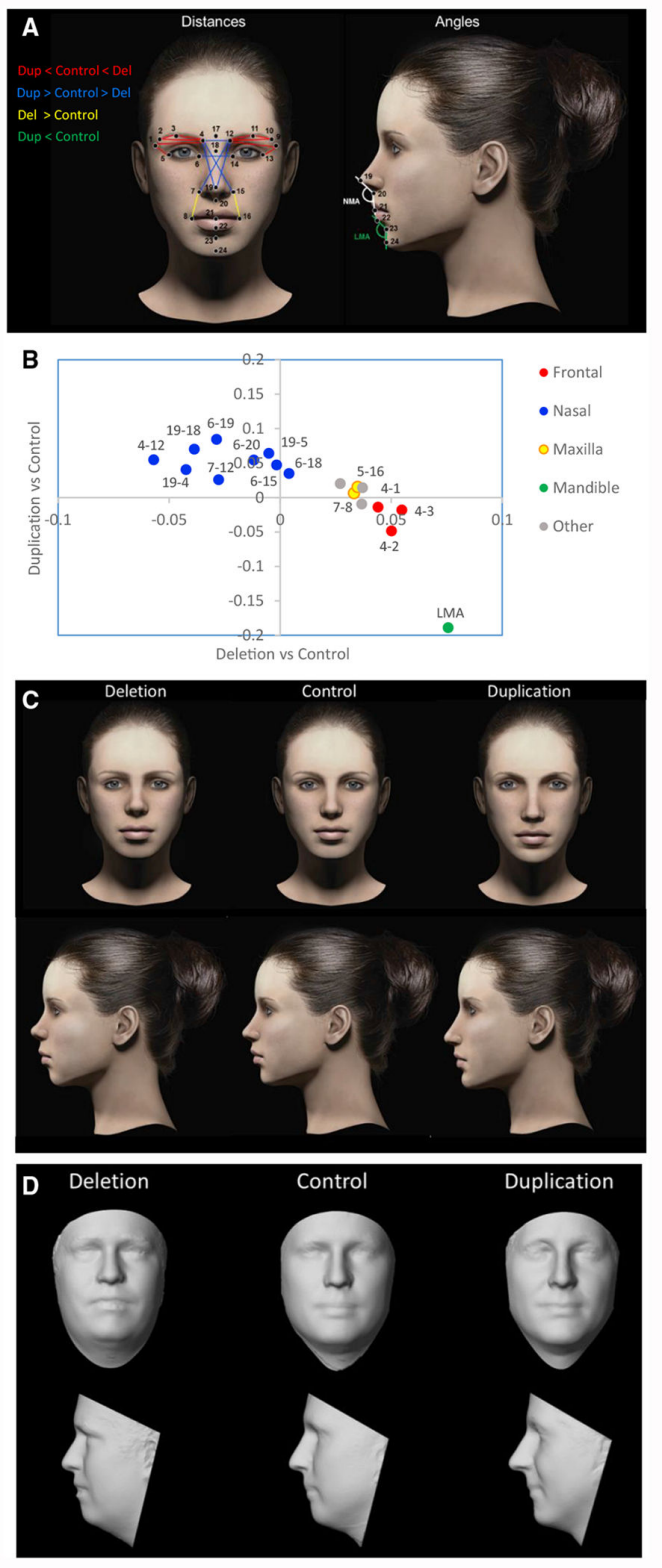
Differential Effects of 16p11.2 Copy Number on Dimensions of the Frontal, Nasal, Maxillary, and Mandibular Regions (A) On each 3D facial image, 24 landmarks were placed and two angular measurements were calculated. A description of landmarks is provided in Table S2. After averaging symmetric distances, 156 distance measures were compared between the CNV and control groups. (B) 18 measures were significant after correction for a FWER <5%. Regression coefficients for duplication versus control (y axis) and deletion versus control (x axis) show that reciprocal CNVs have reciprocal effects on growth of the major craniofacial processes. The category “Other” represents features that span multiple processes. The 14 most informative facial features based on LASSO selection are drawn in (A) and colored by facial region according to the legend. For clarity, some nasal distances are excluded. (C) Facial features associated with deletion and duplication were visualized as a computer-generated model face in which specific features were adjusted according to the observed effect sizes (from B and Table S2). (D) The average surface topography was generated from multiple (>5) age-matched subjects with each genotype. Note that subtle differences in BMI are also apparent; however, these effects are controlled for in the statistical analysis and do not influence the feature selection.

3D morphometric facial imaging was performed on subjects with 16p11.2 duplications or deletions and controls recruited to the Simons VIP study (Simons VIP Consortium, 2012; see STAR Methods). The final dataset (N = 228; Table S1) included 45 with deletions, 44 with duplications, and 139 familial non-carrier controls. A total of 24 landmarks were placed on each image (Figure 1A; descriptions of landmarks are provided in Table S2). “Features,” defined as pairwise distances between landmarks, were normalized to the geometric mean. Differences in deletion and duplication groups relative to controls were detected by linear regression, and covariates were included in the model to control for known factors that influence dimensions of head and face in this genetic disorder, including age, head circumference, BMI, sex, and ancestry principal components obtained from genetic data, with a random intercept allowed to account for within-family correlation.

Eighteen features differed significantly between groups at a family-wise error rate of 5% (Figure 1B; Table S2), and 45 were significant at a Benjamini-Hochberg false discovery rate (FDR) correction of 5%. For 13 of the 18 significant features, deletion and duplication had effects that were opposite in direction (p = 0.048, one-sided binomial test). Consistent with the deletion and duplication having reciprocal effects, the deletion versus duplication effect sizes were negatively correlated for the 18 significant measures (p = < 0.001, Pearson’s correlation = −0.77; Figure 1B).

Genetic effects were clustered in regions corresponding to major processes of craniofacial development (frontonasal, medial nasal, maxilla, and mandible). Deletion of 16p11.2 was associated with significantly larger frontal (4-1, 4-2, 4-3, 12-9, 12-10, and 12-11) and maxillary (7-8, 15-16, 5-16, and 13-8) dimensions and a shorter (18-19) and narrower nose (4-12, 4-15, 6-15, 7-12, and 7-14). By contrast, the duplication was associated with opposite effects, including smaller frontal dimensions (4-2) and significantly wider nose and longer nasal bridge (18-19). Duplications were associated with a narrower labiomental angle (LMA) consistent with a more protrusive chin. A wider LMA was observed in deletion carriers, but the effect did not reach statistical significance in this comparison. Least absolute shrinkage and selection operator (LASSO) logistic regression was performed to select a parsimonious subset of 14 features that could best discriminate each genotype (Table S2; illustrated in Figure 1A).

Facial gestalts associated with the 16p11.2 deletion and duplication were visualized using computer-generated faces in which the features of a model face were adjusted according to the 14 differences described above, including the frontonasal and maxillary distances and the LMA (Figure 1C; Table S2). Dimensions were adjusted based on the percentage difference between CNV and control groups (defined as the effect size divided by the mean). Differences ranged from 1% to 12%.

**Figure 2.**
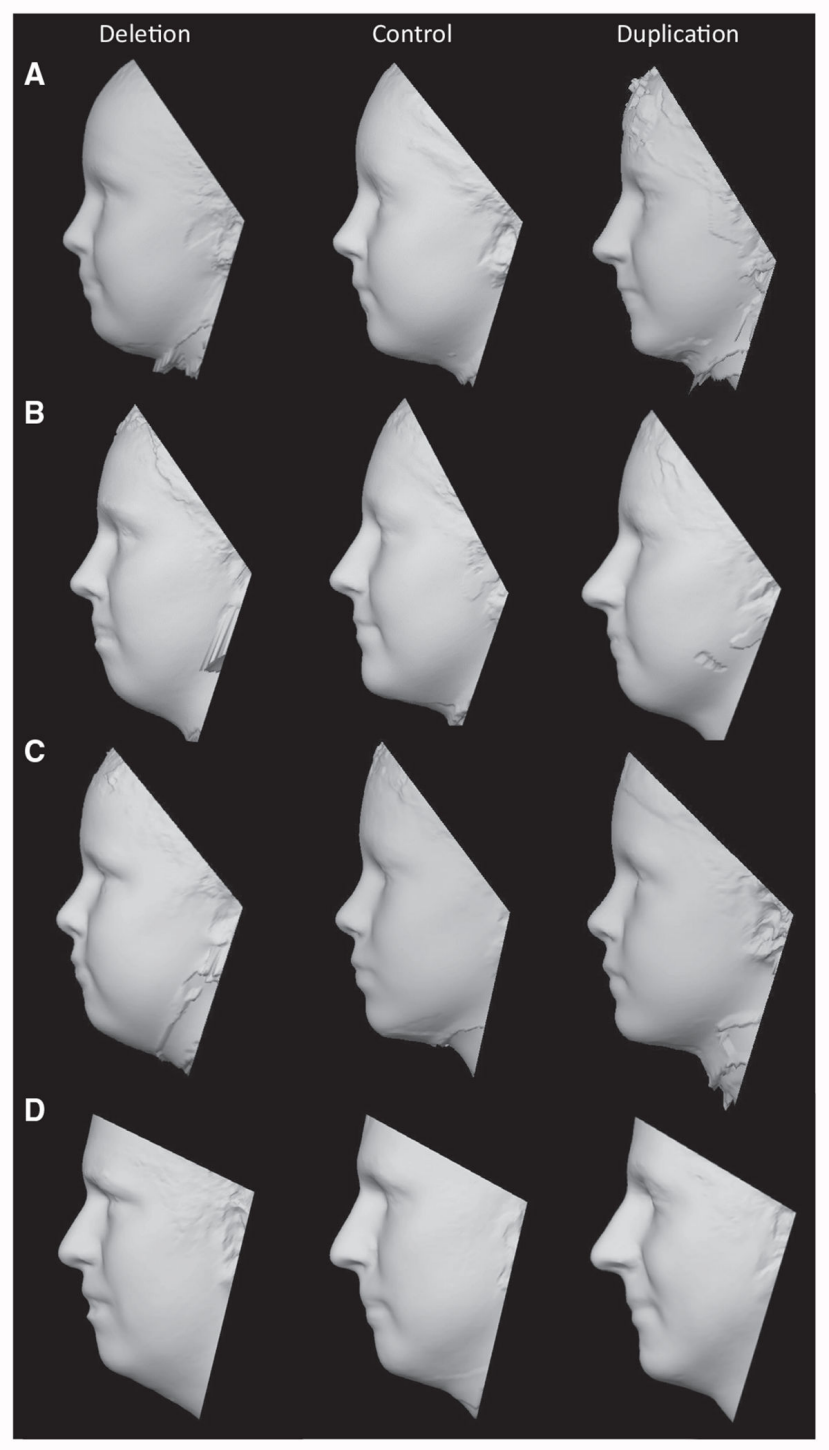
Three-Dimensional Models of Deletion, Control, and Duplication Groups (A–D) 3D models were generated by averaging of the surface topography of faces from multiple subjects. Separate models were constructed for (A) female children (deletion: n = 7, mean age 9.15 years; control: n = 8, mean age 9.92 years; duplication: n = 5, mean age 12.73 years), (B) female adults (deletion: n = 4, mean age 20.13 years; control: n = 8, mean age 23.71 years; duplication: n = 5, mean age 23.25 years), (C) male children (deletion: n = 7; mean age 8.90 years; control: n = 9, mean age 9.27 years; duplication: n = 9, mean age 9.20 years), and (D) male adults (deletion: n = 5; mean age 25.53 years; control: n = 10, mean age 36.59 years; duplication: n = 5, mean age 36.48 years).

To further visualize the facial gestalts of controls, deletion carriers and duplication carriers respectively, a 3D model of each was generated by averaging of the surface topography of faces from multiple subjects (3dMDvultus version 2.5.0.1). The sample was limited to subjects ages 14–49 years to avoid variability in facial features across development at young ages, and the sample was restricted to males for which there were sufficient numbers of age-matched subjects (>3) for all three genotypes (Table S3). A facial gestalt similar to that of the simulated faces was distinguishable in the average faces of adult male deletion and duplication carriers and control subjects, with the effects on the nose and chin being the most recognizable feature (Figure 1D). Similar features were observed in the average faces of younger (ages 8-11 years) and older (ages 18-50 years) subjects of both sexes (Figure 2).

### Craniofacial Characteristics Distinguish 16p11.2 Deletion and Duplication Carriers from Controls

**Figure 3.**
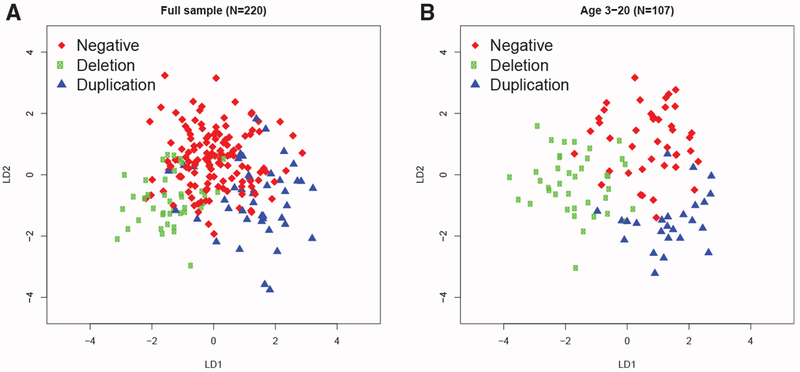
Classification of 16p11.2 Genotype Based on Facial Features (A and B) Discriminant coefficients based on features that were significant at FDR <0.05 can distinguish the subjects based on genotype, with better discrimination for younger subjects (age ≤ 20 years). The linear model was controlled for age, head circumference, BMI, sex, and ancestry principal components. Linear discriminant analysis was applied to subjects for which the above demographic information was complete for the full sample (N = 220; 8 had missing predictors; A) and the younger group (N = 107; 6 had missing predictors; B).

Based on linear discriminant analysis (LDA) of craniofacial features, genotypes could be separated into clusters, with better separation for younger subjects (Figure 3). The LDA model achieved a total correct classification rate of 0.78 on the full sample, reflecting the considerable overlap between the genotypes (Figure 3A). Genotype was classified more accurately by LDA when restricted to younger (ages 3–20 years) subjects (Figure 3B), with total correct classification 0.84. The predictive accuracy of the LDA model was confirmed by leave-one-out cross validation of the full sample, which gave specificities of 0.88 and 0.93 and sensitivities of 0.48 and 0.42 for deletion and duplication, respectively. When restricted to younger subjects, specificities were 0.88 and 0.87, and sensitivities were 0.72 and 0.52 for deletion and duplication respectively.

These results demonstrate that deletion and duplication carriers have combinations of facial features that are distinctive for each group. However, the substantial overlap between the faces of CNV carriers and controls is consistent with many subjects having a non-syndromic appearance that is not characterized by gross anomalies. Examination of group differences on each of the individual distances confirms that deletion and duplication groups do not represent outliers on any single measure (Figure S1).

### Differential Effects of CNV on Craniofacial Structure Are Recapitulated in Rat and Mouse Models of 16p11.2

Rodent models of 16p11.2 deletion and duplication exhibit a variety of behavioral traits (Arbogast et al., 2016; Horev et al., 2011; Yang et al., 2015). However, the direct relevance of these phenotypes to the human condition is uncertain. Similarly, the analysis of anthropometric traits in model organisms has been confounded by growth retardation that is observed in some mouse models (Arbogast et al., 2016; Horev et al., 2011; Yang et al., 2015). We theorized that the cranial skeleton might represent an aspect of vertebrate development that is sufficiently conserved to serve as surrogate traits for genetic dissection of 16p11.2 CNV. To that end, we pursued quantitative analyses of the skull from rat and mouse models of the 16p11.2 deletion and duplication (Arbogast et al., 2016).

Rat deletion and duplication models were generated by CRISPR-Cas9 genome editing of the syntenic region, and computed tomography (CT) scans were obtained from a cohort of 75 rats. In addition, CT scans of mouse lines from Arbogast et al. (2016) were obtained from a cohort of 26 mice (see STAR Methods). For each subject, a set of 19 landmarks were placed delineating the major craniofacial processes, and features were compared between the CNV models and matched controls using linear regression. Results of all univariate tests are described in Table S4.

**Figure 4.**
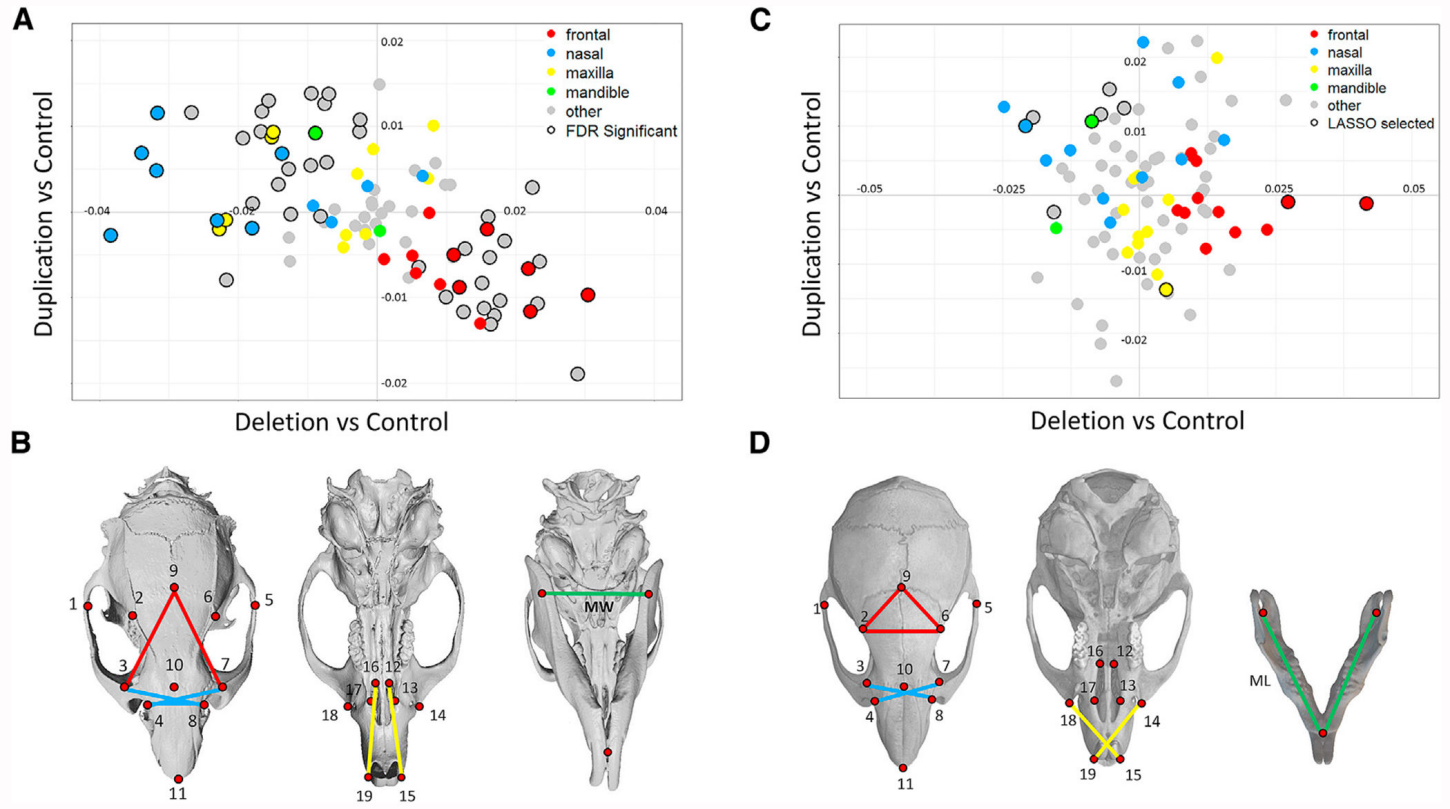
Validation of Mirror Craniofacial Effects in Rat and Mouse Models of 16p11.2 Deletion and Duplication All pairwise distances were analyzed for nineteen landmarks on the dorsal skull and three on the mandible as shown here and in Table S4. Distances are colored according to craniofacial region using the same scheme as in Figure 1. Distances that span multiple craniofacial processes are denoted as “other.” ML, mandibular length; MW, mandibular width. (A) In the rat models, 52 individual features differed significantly by genotype. Regression coefficients for the duplication deletion show significant mirror effects. (B) Informative features were identified by LASSO selection, and features that correspond to a specific facial process in rat are shown. (C) In the mouse models, 12 craniofacial measures that discriminated mutant and control groups were selected by LASSO. Regression coefficients of these features show mirror effects of deletion and duplication similar to those in human and rat. (D) Features that correspond to specific facial processes in mouse.

CNV had a significant effect on craniofacial structure in rat with strong mirror effects across all features between the deletion and duplication models (r = −0.56, p < 0.001; Figure S2). A total of 52 features were significantly associated with genotype (FDR < 0.05; Figure 4A). By labeling features according to their respective craniofacial regions, we observe that the deletion was associated with larger frontal regions (e.g., 9-2, 9-3, 9-6, and 9-7; Figure 4B) and smaller nasal regions (3-7, 3-8, 7-7, and 4-8) and narrower mandibular width (MW), while the opposite effects were associated with the duplication. These results are consistent with the patterns that were observed in human.

Overall the effects of the deletion in mouse were similar to those in rat with effect sizes across the face being significantly correlated between species (r = 0.50, p < 0.0001; Figure S3). The effects of the duplication in mouse did not correlate with those in rat and did not exhibit a strong mirror effect relative to the deletion across all features (p = 0.59), consistent with the duplication having a comparatively modest effect in this mouse line. Mouse craniofacial features that differed between deletion and duplication lines, however, did show mirror patterns similar to those in rat and human (Figure 4C). For the most informative features that were selected by LASSO regression, deletion mice had larger frontal (9-2, 9-6 and 2-6) and maxillary (19-14 and 15-18) distances and smaller nasal features (7-1, 8-1, 8-3, and 10-1) and shorter mandibular length (ML), which were similar to the effects observed in human (sign test p = 0.004), whereas the duplication mouse model had reciprocal effects on the same features (sign test p = 0.004).

### Craniofacial Features Associated with 16p11.2 CNVs Are Attributable to Multiple Genes

**Figure 5.**
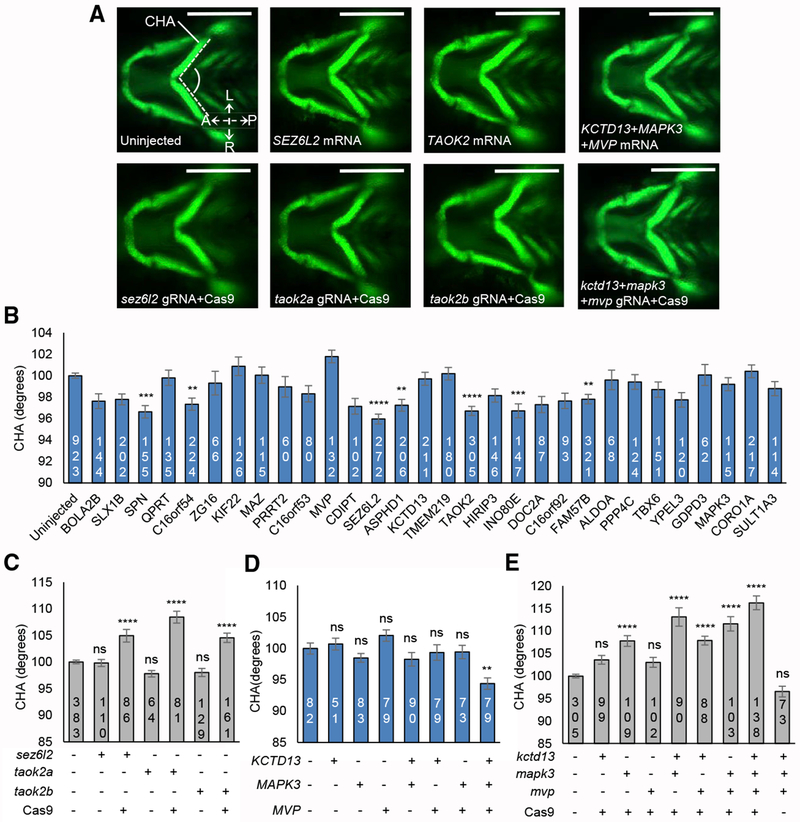
*In Vivo* Modeling of the 16p11.2 CNV Implicates Single Gene Drivers and Epistatic Effects Influencing Cartilage Structures in the Zebrafish Pharyngeal Skeleton (A) Representative ventral images of −*1.4col1a1:egfp* zebrafish larvae at 3 days post-fertilization (dpf). Orientation arrows indicate anterior (A), posterior (P), left (L), and right (R). Scale bar, 300 μm. (B) Quantitative assessment of the CHA of larvae injected with single human mRNAs for each of the 30 genes located in the 16p11.2 BP4-BP5 region. Images were measured as shown in (A) (angle between dashed lines). Seven transcripts induced a significant reduction in CHA after Tukey’s p value adjustment (adjusted p < 0.01). Dosage is 12.5 pg for *KIF22* and *PPP4C* and 50 pg for all other genes. (C) Quantitative assessment of the CHA of F0 mutant batches injected with single combinations of each of *sez6l2*, *taok2a*, and *taok2b* gRNAs with or without Cas9. Dosage is 50 pg gRNA and 200 pg Cas9 protein. (D) Quantitative assessment of the CHA of larvae injected with single or equimolar combinations of human *KCTD13*, *MAPK3*, and *MVP* mRNAs. Dosage is 50 pg. (E) Quantitative assessment of the CHA of F0 mutant batches injected with single or equimolar combinations of *kctd13*, *mapk3*, and *mvp* gRNAs with or without Cas9. Dosage is 50 pg gRNA and 200 pg Cas9 protein. The number of larvae measured for each condition is indicated at the base of each bar in the graphs. The data are represented as the mean ± SEM; ns, not significant; **p < 0.01, ***p < 0.001, and ****p < 0.0001 versus uninjected controls. Tukey’s multiple comparison tests were applied following a significant one-way ANOVA.

To assess with more granularity the influence of the 16p11.2 genes on facial structure, we tested the effects of individual genes on specific craniofacial features that could be measured by *in vivo* imaging of zebrafish larvae. Protrusion of the lower jaw was measured using the ceratohyal arch angle (CHA), where a smaller angle corresponds to a more protrusive jaw and a wider angle corresponds to a receding jaw (Figure 5A). Dimensions of the frontonasal region were measured using the frontonasal area (FNA) and interocular distance (IOD) (Figure S3A); however, we are unable to capture separate frontal and nasal measurements in zebrafish analogous to those in rodent and in human.

We first tested the effect of overexpression of each of the 30 genes in the 16p11.2 region individually, focusing on the lower jaw phenotype, which is more directly analogous to the phenotypes in human and rodent. Consistent with the protruding jaw associated with the duplication in human and rodents, expression of the individual mRNAs resulted in a lower mean CHA relative to controls for a majority of genes tested (24/30, sign test p value < 0.001; Figure 5B). A total of 14 genes had significant negative effects on CHA (unadjusted p < 0.05), and seven genes had highly significant negative effects on CHA (Tukey’s adjusted p < 0.01), including *SPN, C16orf54, SEZ6L2, ASPHD1*, *TAOK2, INO80E*, and *FAM57B*. The genes inducing the most significant phenotypes included *SEZ6L2* (4° decrease in CHA versus controls; Tukey’s adjusted p < 0.0001) and *TAOK2* (3° decrease in CHA versus controls; Tukey’s adjusted p < 0.0001). None of the genes with a positive effect size (increased CHA) were statistically significant.

We next evaluated the effects of ablating endogenous zebrafish *sez6l2, taok2a*, and *taok2b* using CRISPR-Cas9 genome editing and confirmed that the reciprocal loss of these genes results in a reciprocal increase of the CHA in comparison to controls (*Sez6l2* gRNA^+^Cas9 versus controls, 5° increase in CHA, Tukey’s adjusted p < 0.0001; *taok2a* gRNA^+^Cas9 versus controls, 8° increase in CHA, Tukey’s adjusted p < 0.0001; *taok2b* gRNA^+^Cas9 versus controls, 5° increase in CHA, Tukey’s adjusted p < 0.0001; Figures 5A and 5C), consistent with the effect of the deletion.

We showed previously using zebrafish models that overexpression of *KCTD13* individually and in combination with *MAPK3* and *MVP* led to a decrease in head width (Golzio et al., 2012), and knockdown of *kctd13* exhibited mirror effects, a pattern consistent with the human phenotype of the 16p11.2 CNV. We tested overexpression and CRISPR-Cas9 F0 mutants of *KCTD13, MAPK3*, and *MVP* individually and in combinations of two or three genes. Overexpression of the three mRNAs individually did not have a significant effect on CHA, but injection of all three transcripts combined resulted in a significant 6° decrease in CHA relative to controls (Tukey’s p < 0.01; Figures 5A and 5D). Mutants with reciprocal loss of *mapk3* displayed an increased CHA (Figure 5E), and the three-gene combination resulted in a 16° CHA increase (Tukey’s p < 0.0001). Thus, mirror effects of these genes parallel those that are observed in human. We evaluated the body length of larvae injected with a combination of the three guide RNAs (gRNAs) and Cas9 and found no growth retardation compared to controls, supporting further the specificity of the cartilage phenotypes (Figure S4). For FNA and IOD, significant effects were also observed with combinations of two or three genes (Figure S3). Genome editing was associated with reduction in FNA (Figures S3A–S3C), and gene overexpression was associated with increase in IOD (Figures S3D and S3E), results that parallel the effect of the deletion and duplication on nasal regions in human. Evidence for a synergistic effect of MAPK3 in combination with MVP or KCTD13 was observed for dimensions of the frontonasal region, but not the mandible (Table S5). Other combinations were consistent with additive effects (p = 0.99 for additive ANOVA model compared to fully parameterized model). Together, our *in vivo* experiments performed in zebrafish suggest that facial features that are associated with CNV are under the influence of a substantial proportion of 16p11.2 genes, including some that have synergistic effects.

## DISCUSSION

Here, we show that reciprocal CNVs of the 16p11.2 BP4-BP5 region have mirror effects on craniofacial development. Deletion and duplication of 16p11.2 are each associated with facial features that are distinctive; however, both groups overlap with the variability observed in the general population. Dosage of 16p11.2 was associated with a positive effect on nasal and mandibular regions and a negative effect on the frontal regions.

The principal value of these 16p11.2 CNV facial phenotypes are not as a clinical diagnostic markers but rather as a model for studying the genetic mechanisms through which CNVs influence complex traits. Here, we show that mirror effects of CNV on facial features are well conserved in rat and mouse models of 16p11.2 and that the effects of gene dosage on a specific feature (shape of the mandible) can be further modeled in zebrafish. While previous studies have reported mirror effects of CNV on anthropometric traits as well as regional brain volumes in human (Martin-Brevet et al., 2018; Sønderby et al., 2018) and mouse (Horev et al., 2011), there is little direct concordance in the phenotypes between species. The craniofacial phenotypes we describe here are phenotypic features of the 16p11.2 CNV that are demonstrably conserved across model systems.

By investigating individual gene effects on the shape of the mandible in zebrafish, we show that multiple genes within the 16p11.2 region have an influence on craniofacial structure. Thus, the genetic mechanisms through which 16p11.2 CNVs influence development of the head and face are more complex than anticipated from previous studies. We and others have reported that the gene *KCTD13* has a major effect on head size in zebrafish models (Golzio et al., 2012), and deletion of *KCTD13* is associated reduced synaptic transmission in a mouse model (Escamilla et al., 2017). In this study, *KCTD13* expression or ablation in zebrafish embryos did not have significant effects on growth of the mandibular (Figures 4D and 4E) or frontonasal (Figure S4) regions, but effects on these features were detectable when *KCTD13* was tested in combination with the genes MAPK3 and MVP. In light of our present study, *KCTD13* and the factors that are under its regulation, such as RhoA (Lin et al., 2015), could be but one of multiple pathways through which 16p11.2 CNVs influence craniofacial development.

The genes that exhibited the greatest effects on shape of the mandible were *SPN, C16orf54, SEZ6L2, ASPHD1, TAOK2, INO80E*, and *FAM57B*. This set of genes was not clearly distinguishable from the other 23 genes in the region based on their levels of expression in the developing face (Table S6). However, some of these genes have been shown previously to be associated with alterations in head and brain size, such as *TAOK2* (Richter et al., 2019) and *FAM57B* (McCammon et al., 2017). These and other genes within the region function as regulators of cell proliferation and embryonic development (Khosravi-Far et al., 1995). Notably, *TAOK2* is a regulator of mitogen-activated protein kinase (MAPK) signaling (Chen et al., 1999), which is a commonality among multiple 16p11.2 genes, including *MAPK3*, which encodes the extracellular receptor kinase 1 (ERK1) (Meloche and Pouysségur, 2007), and *MVP* (Scheffer et al., 1995), which complexes with ERK2 (Kolli et al., 2004) and regulates ERK signaling (Kim et al., 2006). This pathway-level convergence highlights MAPK signaling as one pathway that may mediate the craniofacial effects that are observed in this study.

The craniofacial features that are associated with the deletion of 16p11.2, including macrocephaly, broad forehead, and underdeveloped nose and chin (micrognathia), bear some similarity to features of monogenic disorders that are caused by mutations in components of RAS-MAPK signaling, such as Noonan (Bhambhani and Muenke, 2014) and cardiofaciocutaneous (CFC) syndromes (Rauen, 1993). Similar craniofacial anomalies are also observed in mouse embryos with conditional disruption of MAPK signaling in neural crest cells (Parada et al., 2015). Common facial features between 16p11.2 deletions and a subset of these other syndromes is intriguing and suggests that dysregulation of RAS-MAPK signaling might affect embryonic patterning in similar ways in 16p11.2 microdeletion syndrome and the family disorders known as the “rasopathies” (Araki et al., 2004).

An oligogenic mechanism is unlikely to be unique to the 16p11.2 locus. Rather an oligogenic model may apply in general to the effect of large CNVs on complex traits. For example, the polygenic contribution to height appears to be distributed across a large proportion of the genome (Boyle et al., 2017; Liu et al., 2018). The same is likely to be true for other anthropometric and cognitive traits such as facial features, body mass, and IQ. In principle, haploinsufficiency of any set of 30 adjacent genes could impact a variety of complex traits. The features that are prominent for a particular CNV could be those traits for which the CNV gene set has a strong net effect.

Previous studies have found evidence that multiple genes within the 16p11.2 region impact various aspects of development in zebrafish (McCammon et al., 2017) and *Drosophila* (Iyer et al., 2018). However, a major limitation has been a lack of validation of these phenotypes as models of the human disorder. The reciprocal craniofacial phenotypes that we observe are human 16p11.2-associated traits that are reproducible across multiple model organisms, both in magnitude and direction of effect. The skeletal phenotypes that we describe in zebrafish could be useful models for characterizing the additive or epistatic effects of multiple 16p11.2 genes. However, some caution is warranted in interpreting how effects observed in fish relate to the effects of the full CNV. More precise determination of the contribution of individual genes or combinations of genes to the phenotype of the large CNV will require that we return to the rodent models to validate specific gene interactions.

Knowledge of the influence of 16p11.2 deletion and duplication on craniofacial development could serve as a guide for how these genetic disorders influence embryonic patterning more broadly, including regional patterning of the brain (Chang et al., 2016; Owen et al., 2014,^2018^; Qureshi et al., 2014). Further studies of the oligogenic effects described here could provide insights into mechanisms underlying cognitive impairments of these genetic disorders.

## STAR★METHODS

### LEAD CONTACT AND MATERIALS AVAILABILITY

Mouse lines are available through the INFRAFRONTIER repository European Mouse Mutant Archive (EM:06133 and EM:06134). Rat lines will also be deposited into the INFRAFRONTIER repository and in the interim can be obtained by request from Yann Herault. Further information and requests for resources and reagents should be directed to the Lead Contact, Jonathan Sebat (jsebat@ucsd.edu).

### EXPERIMENTAL MODEL AND SUBJECT DETAILS

#### Human subjects

Subjects were recruited in conjunction with the Simons VIP study (Simons VIP Consortium). 3D morphometric facial imaging was performed on a subset (N = 359) of subjects with 16p11.2 duplications or deletions from the Simons VIP cohort at 3 sites (University of Washington, Harvard University, and Baylor College of Medicine) using the 3DMD 3-pod camera system. Data analysis was restricted to subjects of European ancestry older than 3 years of age. Additional subjects were excluded due to facial hair, image quality or landmark visibility (i.e., obscured by clothing, hair or makeup). The final dataset (N = 228) included 45 deletions, 44 duplications and 139 familial non-carrier controls (Table S1).

#### Rodent models of 16p11.2 deletion and duplication

The 0.5 Mb region of human 16p11.2 that is commonly deleted or duplicated in these genetic disorders is highly syntenic (same genes in the same order) to chr1:198,100,000-198,583,000 of the rat genome (RatRnor_6.0) and to the orthologous0.5 Mb region of mouse chr7F4 (See Arbogast et al.).

The mouse models of 16p11.2 used in this study consisted of deletion (*Del*/+) or duplication (*Dup*/+) of the *Sult1a1-Spn* genetic interval (Arbogast et al.) Lines were maintained on a pure C57BL/6N C3B genetic background. Rat deletion and duplication models were generated by CRISPR/Cas9 genome editing of Sprague Dawley line (Charles River Laboratory, Oncins, France). Briefly a deletion of 483,122 bp located at positions chr1:198,100,544-198,583,667 (RatRnor_6.0) and a duplication of the interval from chr1:198,100,545-198,583,458 (RatRnor_6.0), corresponding to the 16p11.2 homologous region of the rat genome, were obtained. For the genotyping, primer pairs were designed for the Del, Dup and an internal control alleles (Primers Del: rHamont99For: GGGCTGGCAGACTTGAA

rHavalB284Rev: GTGCCACGATCAGCAG; Primers Dup: rHamont99Rev: CGCTTTGATGCCCACTA; rHavalB84For: AGCTGTGA TCCTCTGGTT; Primers for internal control: rAnks3-205For: CCCCAGCCTCCCACTTGTC, rAnks3-205Rev: AGGATGACT GAAATTGGTGGAC) to amplify specific PCR fragments (Del: 290bp, Dup: 500bp, internal control 205bp) using standard conditions (Roche, 60°C for primer hybridation).

A cohort of 75 rats was bred for craniofacial analysis, which included 23 *Del*/+ (9 male and 14 female), 26 *Dup*/+ (13 male and 13 female) and 26 +/+ siblings (13 male and 13 female). The mouse models of 16p11.2 used in this study consisted of deletion (*Del*/+) or duplication (*Dup*/+) of the *Sult1a1-Spn* genetic interval (Arbogast et al., 2016) Lines were maintained on a pure C57BL/6N C3B genetic background. A mouse cohort was bred including 36 females at 13 weeks of age, including 10 *Del*/+ and 10 +/+ littermates, and 8 *Dup*/+ and 8 +/+ littermates.

#### Zebrafish lines

Zebrafish embryos were obtained from natural matings of heterozygous *−1.4col1a1:egfp* transgenic adults maintained on an AB background (Kague et al., 2012).

#### METHOD DETAILS

##### 3D Morphometric Analysis of Simons VIP subjects

The goal of this study was to define specific craniofacial features in human that are influenced by 16p11.2 copy number and to subsequently validate the observed effects in animal models of the 16p11.2 CNV. To this end, we sought capture specific dimensions of the major skeletal processes in human that can also be captured by computed tomography (CT) scans of rodents including frontal, nasal, maxillary and mandibular regions. For this purpose, regression-based analysis of a defined set of linear or angular features is preferable to dense surface modeling-based approaches (Hammond et al., 2014) that are more optimal for capturing a wide variety of facial features including subtle effects on surface curvature.

Images of Simons VIP subjects were acquired using the 3dMDtrio system (http://www.3dmd.com). A total of 24 landmarks were placed blind to genotype, including 20 that were landmarked according to Farkas standards (Farkas, 1994) and four additional landmarks that were placed to capture frontal dimensions including lateral brow (landmarks 2 and 10) and medial brow (4 and 12). A visualization (Figure 1A) and description (Table S2) of the 24 landmarks is provided.

##### Rodent skull Imaging and Landmarking

For both rat and mouse cohorts, images of the dorsal skulls were captured using a microCT imaging system (Quantum GX, Perkin Elmer, France). For rats, an image was acquired for the complete skull. For mice, images of the dorsal skull and lower jaw of each animal were acquired separately as part of a previous study (Arbogast et al.). Nineteen landmarks were placed representing the frontal, nasal and maxillary regions, and all pairwise distances between landmarks were normalized to the geometric mean. In addition the mandibular length (ML) and width (MW) were determined by first determining the centroid of multiple landmarks at the lower incisors and the left and right ramus (Figure S5), and then determining distances between the three centroids. Symmetric distances of the skull and mandible were averaged.

##### mRNA overexpression and CRISPR/Cas9 genome editing in zebrafish embryos

To model the 16p11.2 BP4-BP5 duplication, we overexpressed individually each gene of the region (see Figure 5B). We linearized pCS2+ constructs (Golzioet al., 2012) and transcribed human mRNA using the mMessage mMachine SP6 Transcription Kit (Ambion). All RNAs were injected into the yolk of the embryo at the 1- to 4-cell stage at 50, 25, or 12.5 pg doses (1 nl/injection). To investigate specific gene interactions that have been reported previously (Golzio et al.), *KCTD13, MAPK3*, and *MVP* mRNAs were tested in combinations of two or three. Two way and three way gene interaction models were fitted to test the synergy effect from double-hit or triple-hit groups. Packages “multcomp” from R (version 3.4.1) was used.

CRISPR/Cas9 genome editing was performed as a model of the reciprocal deletion. We used CHOPCHOP(Labun et al., 2016) to identify guide (g)RNAs targeting coding sequence within *kctd13, mapk3, mvp, sez6l2, taok2a*, and *taok2b*. Primers sequences are provided in Table S7 and experimental validation of mutant lines is provided in Figure S7. Briefly gRNAs were transcribed *in vitro* using the GeneArt precision gRNA synthesis kit (ThermoFisher) according to the manufacturer’s instructions; 1 nL of injection cocktail containing 50 pg/nl gRNA and 200 pg/nl Cas9 protein (PNA Bio) was injected into the cell of embryos at the 1-cell stage. To determine targeting efficiency in founder (F0) mutants, we extracted genomic DNA from 2 day post-fertilization (dpf) embryos and PCR amplified the region flanking the gRNA target site. PCR products were denatured, reannealed slowly and separated on a 20% TBE 1.0-mm precast polyacrylamide gel (ThermoFisher), which was then incubated in ethidium bromide and imaged on a ChemiDoc system (Bio-Rad) to visualize hetero- and homoduplexes. To estimate the percentage of mosaicism of F0 mutants (n = 5/condition), PCR products were gel purified (QIAGEN), and cloned into a pCR8/GW/TOPO-TA vector (Thermo Fisher). Plasmid was prepped from individual colonies (n = 9-12 colonies/embryo) and Sanger sequenced according to standard procedures.

##### Automated zebrafish imaging

Larvae were maintained under standard conditions at 28.5°C until 3 dpf and were positioned and imaged live as described (Isrie et al., 2015). Automated imaging was conducted with an AxioScope.A1 microscope and Axiocam 503 monochromatic camera facilitated by Zen Pro software (Zeiss), to capture dorsal images of GFP signal. Larval batches were positioned and imaged live using the Vertebrate Automated Screening Technology (VAST; software version 1.2.5.4; Union Biometrica) BioImager. Larvae from each experimental condition were anesthetized with 0.2 mg/mLTricaine prior to being loaded into the sample reservoir. Dorsal and lateral image templates of uninjected controls and experimental larvae were created and we acquired images at a > 70% minimum similarity for the pattern-recognition algorithms. Larvae were rotated to 180° to acquire ventral images via a 10x objective and fluorescent excitation at 470nm to detect GFP to capture fluorescent images of the pharyngeal skeleton. ImageJ software (NIH) was used to measure the angle of the ceratohyal cartilage. All experimental conditions were normalized to uninjected controls and set to 100 degrees. Statistical comparisons were performed using one-way ANOVA with Tukey’s test (GraphPad Prism).

##### Examining levels of gene expression during murine craniofacial development

We examined whether the significant effects of seven genes (*SPN, C16orf54, SEZ6L2, ASPHD1, TAOK2, INO80E and FAM57B*) on shape of the mandible could be attributable to the differential regulation of these genes. A published dataset was obtained consisting of Affymetrix (Mouse Gene ST 1.0 array) gene expression analysis of the major craniofacial processes of the developing mouse embryo (E10.5-E12.5) (Hooper etal., 2017) (accession # FB00000803, Facebase.org). Samples included mesenchymal and ectodermal cells of the frontonasal and mandibular processes of embryos at E10.5, E11.5 and E12.5 in triplicate, and samples of the maxillary process at E11.5 and E12.5. Summary gene level expression data was obtained (https://www.facebase.org/hatrac/facebase/data/ fb2/FB00000803/rma.summary.names.txt) and the basal expression levels of the seven 16p11.2 genes identified in this study (see Figure 4) was compared to the levels of the other twenty-two 16p11.2 genes in each structure was determined by averaging the expression values across replicates and embryonic stages. Results show the expression levels of all three genes to be consistent across cell types and structures of the face, and the seven genes were not expressed at higher levels on average than the other twenty two (Table S6).

#### QUANTIFICATION AND STATISTICAL ANALYSIS

##### Analysis of human craniofacial features

Quantitative measurement of all pairwise distances between 24 landmarks were calculated using the 3dMDvultus - Analysis software, version 2.5.0.1 (http://www.3dmd.com). Symmetric distances were averaged, yielding 156 facial distance measurements. Each distance was normalized to the overall size of the individual’s face, by dividing by the geometric mean of the 156 distances for that individual. Angular measurements of the nose and chin, the nasomental (NMA) and labiomental (LMA) angles respectively, were calculated by triangulating the relevant landmarks. A series of linear mixed-effects models, using package lme4 in R (version 3.4.1), was used to separately test for the effect of deletion and of duplication on each angle or normalized facial distance. Each model controlled for fixed effects of age, head circumference, body mass index (BMI), sex, and ancestry principal components, with a random intercept allowed to account for within-family correlation. Interaction between genotype and sex was included if significant at 5% level. Significant differences according to genotype were determined by a likelihood-ratio test at a family-wise error rate of 5% using Holm’s correction (Holm, 1979).

##### Controlling for variation in ancestry

All subjects were of European ancestry, however regional genetic differences could still explain variation in facial traits. We controlled for ancestry using principal components derived from genetic data subjects. Ancestry principal components were obtained on 213 subjects from Illumina SNP genotype data (Illumina HumanOmniExpress v.1 and v.2) available from the Simons VIP study (www.sfari.org/resource/simons-searchlight/). Missing data on 38 subjects was imputed by using PCs from a sibling nearest in age or a randomly selected parent. After imputation, 15 subjects from two different families were still missing data, 9 of which were familial controls and 6 were duplications. Analyses were performed with and without including the ancestry principal components as a sensitivity analysis, and the results were very similar. Significant correlation was observed between one of the first two principal components and 7 of the 160 craniofacial distances.

##### Generating 3D models by averaging faces of deletion, duplication and control subjects

To visualize the respective facial gestalts of controls, deletion carriers and duplication carriers, a 3D-model of each was generated by averaging of the surface topography of faces from multiple subjects using the 3dMDvultus-Analysis software, version 2.5.0.1 (www.3dmd.com). To maximize the number of unrelated subjects that were closely matched in age within each group, selection criteria for averaging of faces differed slightly from that of the overall dataset. Only unrelated individuals were included, additional subjects were removed due to image quality (gaps in the surface topography), and the requirement for landmark visibility was relaxed, allowing for frontotemporal landmarks (landmarks 1 and 9, see Figure 1) to be covered in some cases by hair or headwear. We first restricted our 3D models to young adults (age 14) and older to avoid variability in facial features across development at young ages, and the sample was restricted to males (the largest group). Subjects consisted of 5 Deletion carriers (average age 25.5 years), 5 duplication carriers (36.5 years) and 10 controls (36.6 years). Four Landmarks (the Exocanthion, Glabella and Subnasale, Figure 1. Landmarks 5, 13, 17, 20) were placed manually, and the software’s average-face function was used to generate the average face. The surface property of the 3D image was then converted from a photographic image into a textured-model (Figure 1D). Subsequently, to determine if similar facial gestalts are apparent for other demographics, additional 3D models were generated for children and females (Figure 2). Subjects that were included in average face models are listed in Table S3.

##### Linear Discriminant Analysis

Linear discriminant analysis (LDA) was performed on linear (mixed) model residuals after adjusting for age, head circumference, body mass index (BMI), sex, and ancestry principal components, for both total subjects and subjects aged < 20 years (A random intercept to account for within-family correlation was included when significant). The 45 distances with FDR q value less than 0.05 were used. We used the function “lda” from the “MASS” package in R (version 3.4.1). Specificity and sensitivity were calculated based on LDA prediction with default values.

##### Least absolute shrinkage and selection operator (LASSO) logistic regression

Generalized linear model (logistic regression) with lasso was performed for both human subjects and mice. Distances with FDR q value less than 0.05 were used for human subjects, while distances with a statistically significant likelihood ratio test at p < 0.05 were used for mouse skulls. We used 10-fold cross validation for lasso with minimum deviance for human subjects, and lasso with minimum Akaike Information Criteria (AIC) for mouse skulls (Akaike, 1974). Specificity and sensitivity were calculated based on lasso selected models. Packages “glmnet” and “glmpath” from R (version 3.4.1) were used.

##### Rodent craniofacial analysis

Differences in facial features between deletion and control lines and differences between duplication and control lines were tested with univariate linear models. We tested all 91 distances on the dorsal skull and two distances on the mandible. Effects that were significant at an FDR of 5% were identified. In addition, as we did previously in human, we identified a set of features that distinguish CNV models from controls by performing least absolute shrinkage and selection operator (LASSO) based on all univariate significant distances by generalized linear model, with AIC as the criteria rule.

#### DATA AND CODE AVAILABILITY

Human 3D images and were obtained from the Simons VIP Consortium (Simons VIP Consortium, 2012). Clinical phenotypes and SNP and CNV genotype data on Simons VIP subjects were obtained from SFARI base (https://base.sfari.org/). Quantitative data extracted from 3D surface images and CT scans of human, rat, and mouse are available by request from Jonathan Sebat (jsebat@ucsd.edu).

## Supplementary Material

Supplemental Information

Table S1

Table S2

Table S3

Table S4

Table S5

Table S6

Table S7

## Figures and Tables

**Table:** KEY RESOURCES TABLE

REAGENT or RESOURCE	SOURCE	IDENTIFIER
Deposited Data		
3DMD image data on Simons VIP subjects	Simons VIP Consortium	Not applicable
Genotype data on Simons VIP subjects	Simons Foundation Autism Research Initiative (SFARI)	https://base.sfari.org/
Experimental Models: Organisms/Strains		
Mouse: 16p11.2 Del	European Mouse Mutant Archive	EM:06133
Mouse: 16p11.2 Dup	European Mouse Mutant Archive	EM:06134
Rat: 16p11.2 Del	European Mouse Mutant Archive	pending
Rat: 16p11.2 Dup	European Mouse Mutant Archive	pending
Zebrafish: – 1.4col1a1:egfp	Kague et al., 2012	–1.4col1a1:egfp

## Highlights

- Craniofacial effects of the 16p11.2 CNV are recapitulated in rat and mouse models
- Deletion and duplication have mirror effects on craniofacial features in humans
- Facial features associated with the CNV are attributable to multiple genes
