# Supplementary material for: Oligogenic Effects of 16p11.2 Copy-Number Variation on Craniofacial Development
Source: Cell Rep. Author manuscript; Available in PMC 2020 Jan 29. (PMC6988705; doi:10.1016/j.celrep.2019.08.071)

Cell Reports, Volume *28*

Supplemental Information

Oligogenic Effects of 16p11.2 Copy-Number Variation on Craniofacial Development

Yuqi Qiu, Thomas Arbogast, Sandra Martin Lorenzo, Hongying Li, Shih C. Tang, Ellen Richardson, Oanh Hong, Shawn Cho, Omar Shanta, Timothy Pang, Christina Corsello, Curtis K. Deutsch, Claire Chevalier, Erica E. Davis, Lilia M. Iakoucheva, Yann Herault, Nicholas Katsanis, Karen Messer, and Jonathan Sebat

Figure S1. Craniofacial features that distinguish deletion, duplication and control groups, related to Figure 2. Eighteen craniofacial measures differed significantly between CNV carriers and controls. Box plots illustrate the median, quartiles and a range of 1.5 times the IQR beyond upper and lower quartiles (whiskers) across the full dataset.

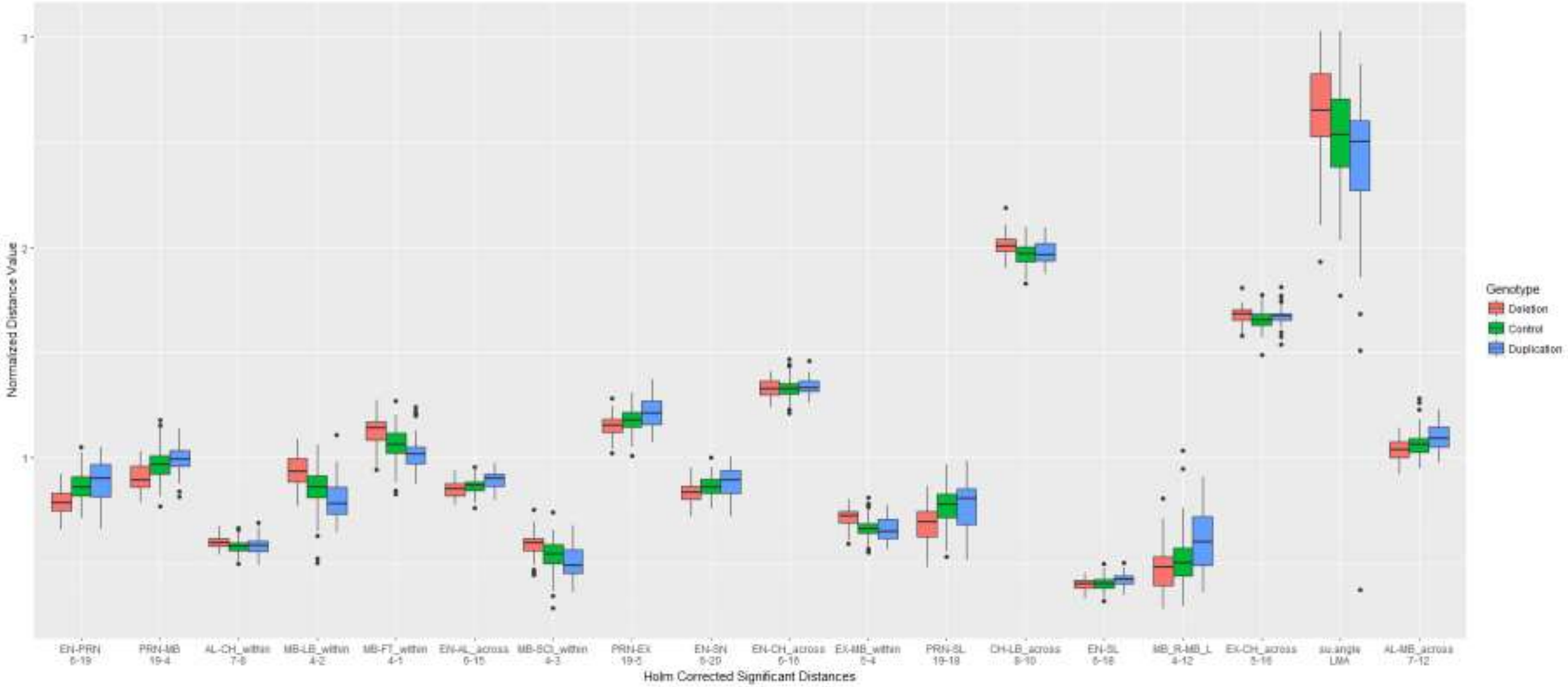

Figure S2. Comparison of the craniofacial effects of CNV in rat and mouse, related to Figure 3. Significant mirror effects of the deletion and duplication are observed in rat across all facial features based on the correlation of the regression coefficients across all features. A significant correlation of facial measures was also observed between the rat and mouse models of the deletion. The effects of the duplication in mouse did not show a significant correlation with the other rodent models.

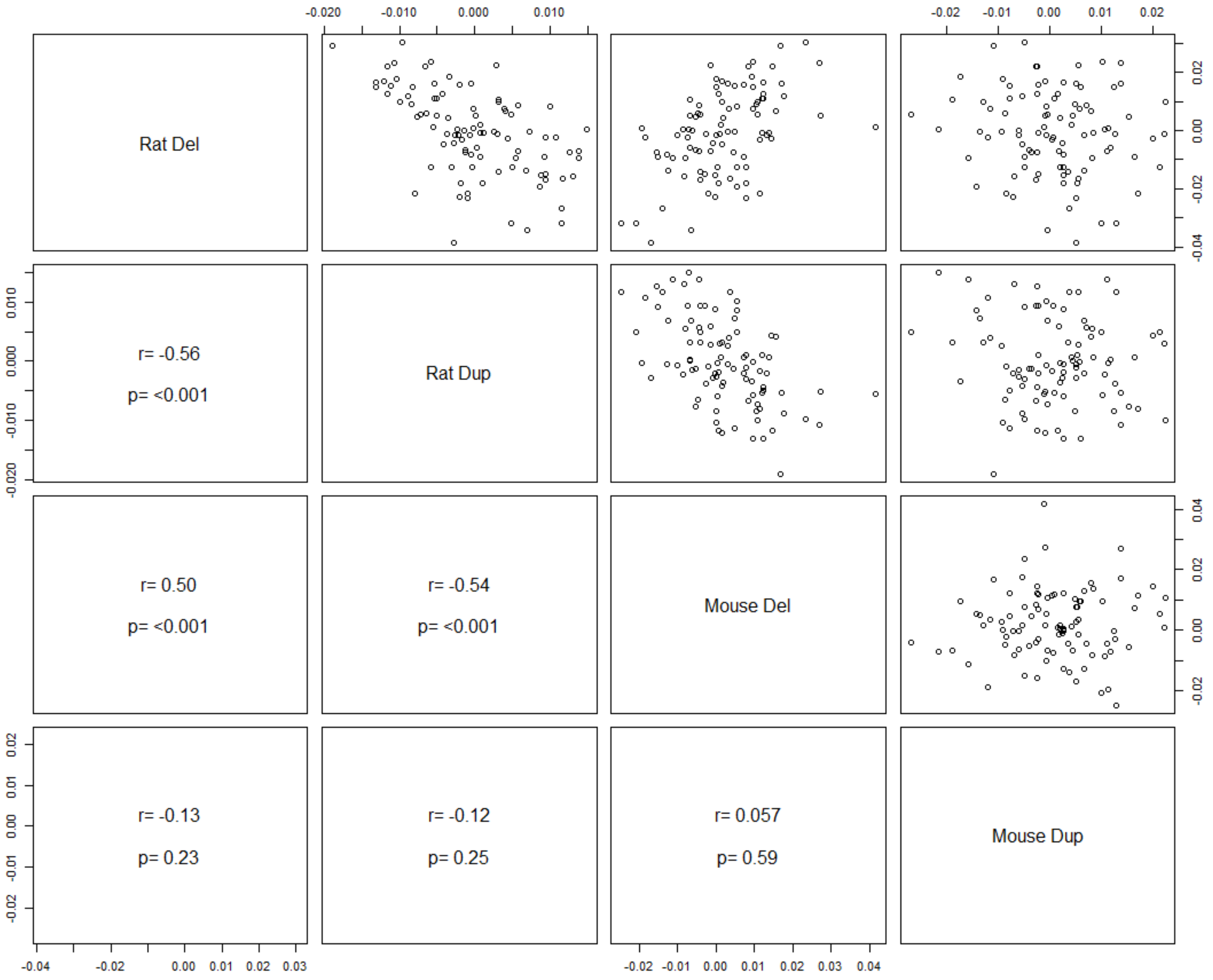

Figure S3. *KCTD13*, *MAPK3*, and *MVP* dose combinatorial suppression result in a decreased frontonasal area whereas similar overexpression results in an increased interocular distance, related to Figure 4. (**A**) Representative ventral images of *-1.4col1a1:egfp* zebrafish larvae at 3 days post-fertilization (dpf). Orientation arrows indicate anterior (A), posterior (P), left (L) and right (R). Area between the eyes: dashed white line. Interocular distance: red line. Scale bar, 200 µm. (**B, D**) Quantitative assessment of the frontonasal area. (**C, E**) Quantitative assessment of the interocular distance. The data are represented as the mean ±s.e.m.; ns=not significant; *P<0.05, **P<0.01 and ****P<0.0001 vs uninjected controls. Tukey's multiple comparison tests was applied following a significant one-way ANOVA

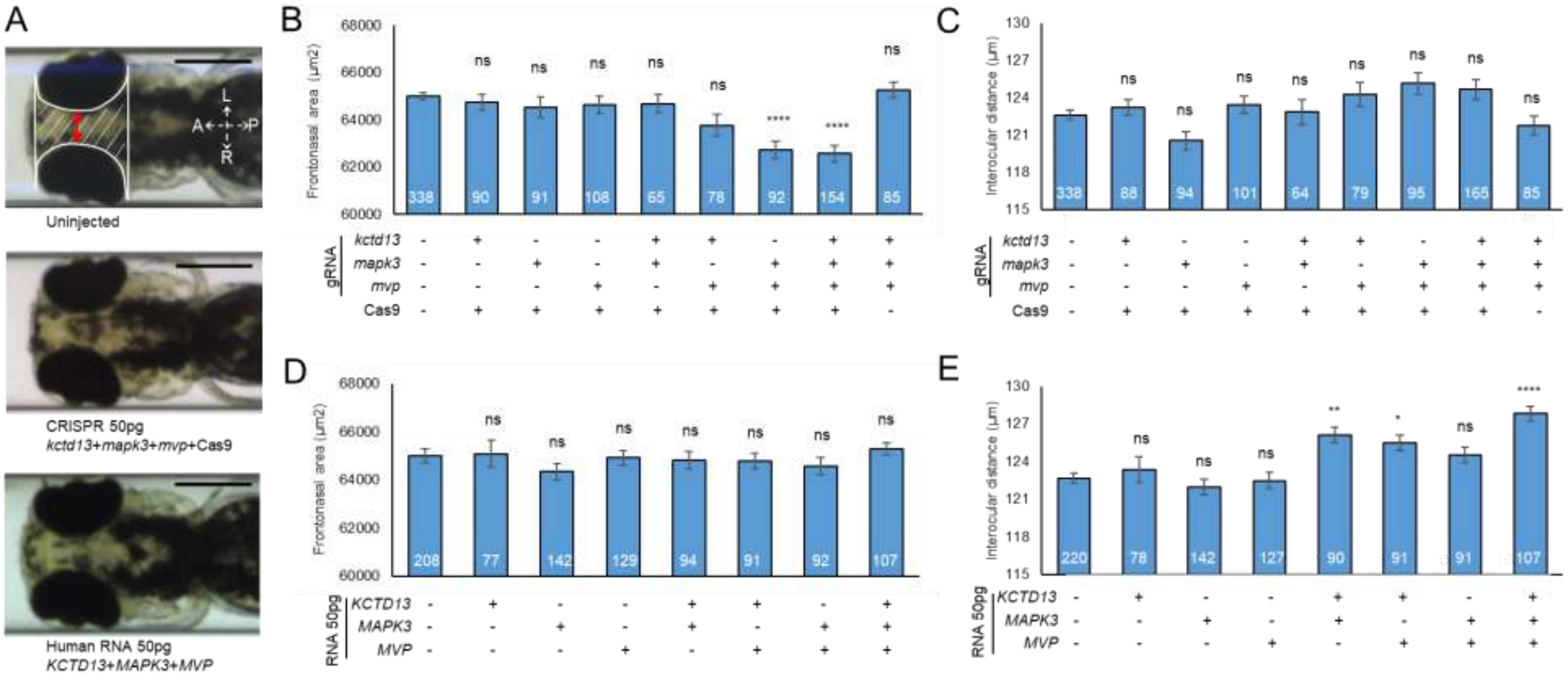

Figure S4. *kctd13*, *mapk3*, and *mvp* dose combinatorial suppression do not induce growth delay, related to Figure 4. (**A**) Representative lateral images of *-1.4col1a1:egfp* zebrafish larvae at 3 dpf. Measurement of the body length is shown with a white dashed line. Orientation arrows indicate anterior (A), posterior (P), dorsal (D) and ventral (V). Scale bar, 600 µm. (**B**) Scatterplot of all larvae; x-axis, area between the eyes; y-axis, body length. Each dot corresponds to one larva. F0 mutant larvae injected with equivalent amounts of *kctd13*, *mapk3*, and *mvp* guide demonstrate a reduction in frontonasal area (as also described in figure 4) but this effect does not correspond to a reduction of overall body length.

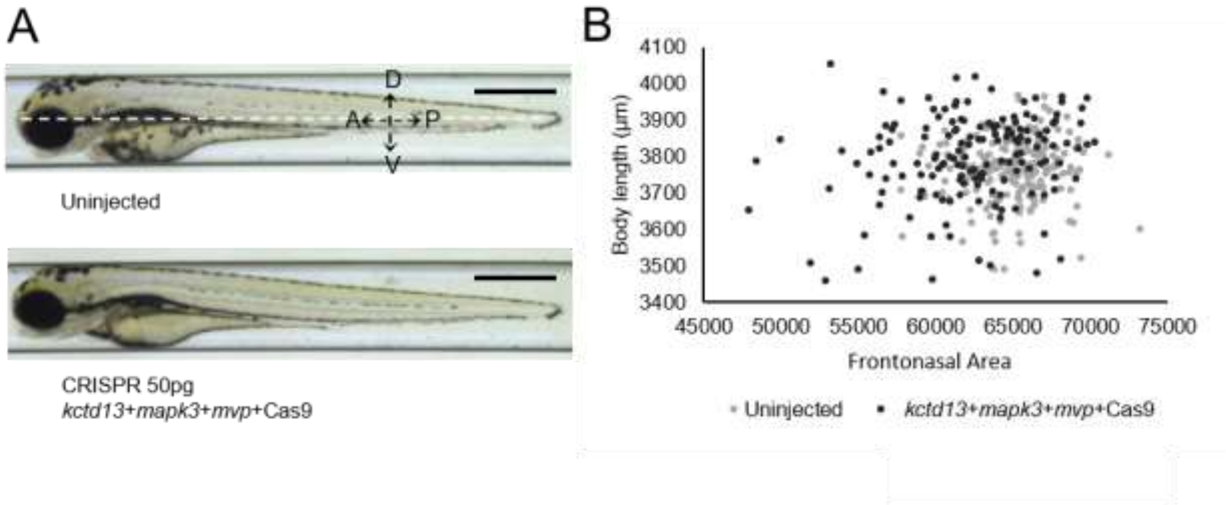

Figure S5. Landmarks that were used to determine the length and width of the mouse mandible, related to STAR methods section "Rodent skull imaging and landmarking". (A) On each jaw bone**,** two posterior landmarks were placed above and below the incisors (yellow) and four anterior landmarks were placed along the ramus (blue = left, red = right). **(B)** Landmarks were grouped by color and the centroid of each group was calculated from X,Y,Z coordinates. The distances between the three centroids was then used to determine the mandibular width (MW) and the length of the left (ML-L) and right (ML-R) mandible.

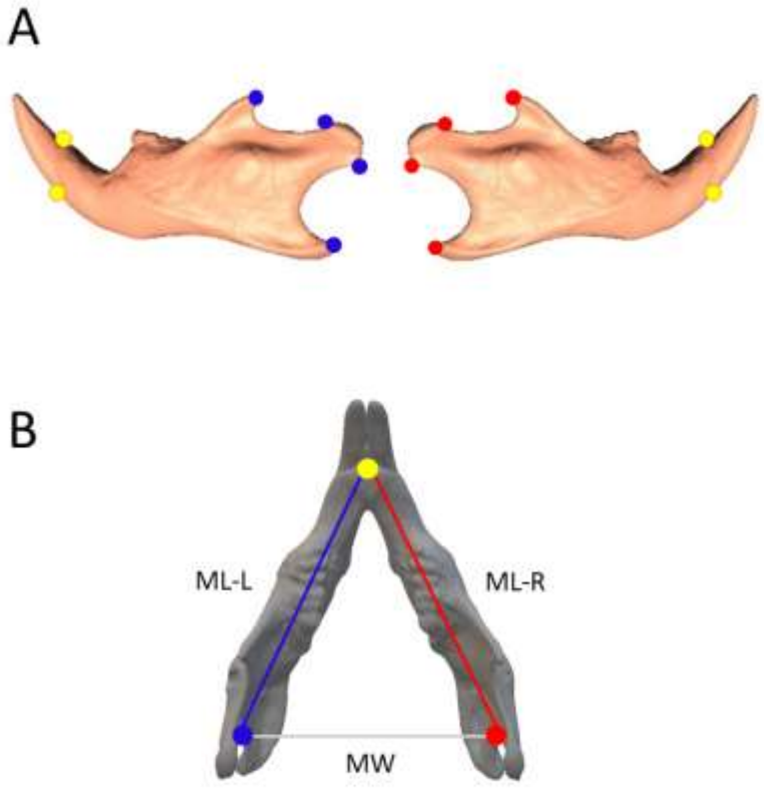

Figure S6. CRIPSR/Cas9 genome editing to generate F0 zebrafish mutants, related to STAR methods section "mRNA overexpression and CRISPR/Cas9 genome editing in zebrafish embryos". Schematics are provided for each of the zebrafish orthologues (**A**) *MAPK3*, (**B**) *MVP*, (**C**) *KCTD13*, (**D**) *SEZ6L2* (**E**) *TAOK2A* and (**F**) *TAOK2B*. A map of each gene locus is illustrated with exons (black boxes), untranslated regions (white boxes), introns (dashed lines), and guide (g)RNA target site and primers used red box and triangles, respectively). Assessment of genome-editing efficiency using polyacrylamide gel electrophoresis (PAGE) is shown. Genomic DNA was extracted from single embryos at 2 dpf, and PRC amplified. PCR products were denatured, reannealed slowly and migrated on a 20% polyacrylamide gel. All twelve F0 embryos displayed heteroduplexes not present in two uninjected controls. Asterisks (*) indicate embryos assessed for percent mosaicism with PCR8/GW/TOPO-TA cloning and Sanger sequencing of individual clones. Representative sequence alignments are shown illustrating the most common targeting events for each embryo. Some clones harbored deletions (green) and some harbored insertions (blue), suggesting ~100% efficiency. gRNA sequence (gray) and protospacer adjacent motif (PAM, red) are shown.

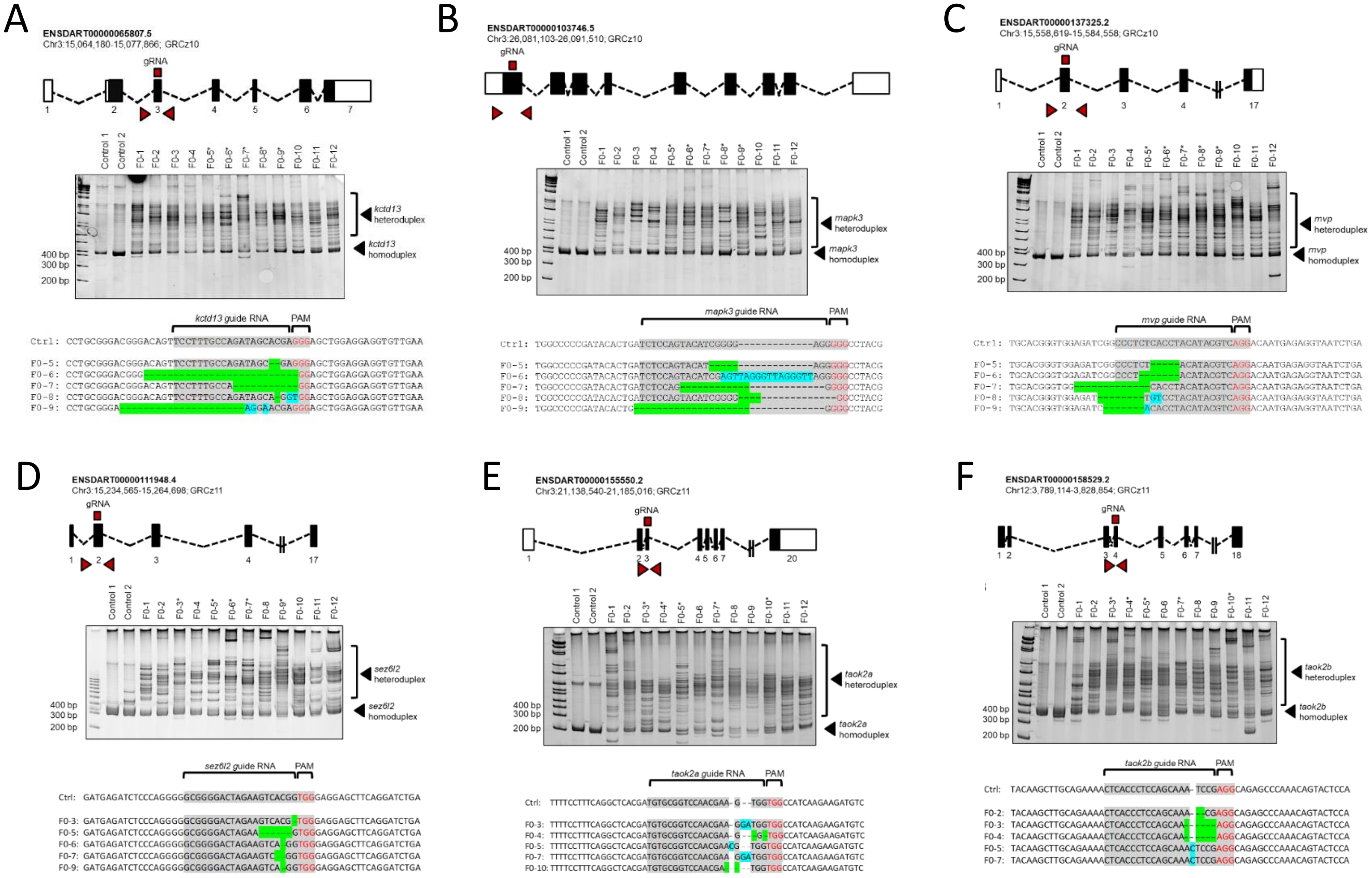

Supplement: Supplemental Information [file NIHMS1543624-supplement-Supplemental_Information.pdf]
